# Tetrahydroxylated bile acids improve cholestatic liver and bile duct injury in the *Mdr2^−/−^* mouse model of sclerosing cholangitis via immunomodulatory effects

**DOI:** 10.1002/hep4.1998

**Published:** 2022-06-12

**Authors:** Claudia D. Fuchs, Emmanuel D. Dixon, Tim Hendrikx, Veronika Mlitz, Annika Wahlström, Marcus Ståhlman, Hubert Scharnagl, Tatjana Stojakovic, Christoph J. Binder, Hanns‐Ulrich Marschall, Michael Trauner

**Affiliations:** ^1^ Hans Popper Laboratory of Molecular Hepatology, Division of Gastroenterology and Hepatology, Department of Internal Medicine III Medical University of Vienna Vienna Austria; ^2^ Department of Laboratory Medicine Medical University of Vienna Vienna Austria; ^3^ Department of Molecular Genetics Maastricht University Maastricht the Netherlands; ^4^ Department of Molecular and Clinical Medicine Sahlgrenska Academy University of Gothenburg Gothenburg Sweden; ^5^ Clinical Institute of Medical and Chemical Laboratory Diagnostics Medical University of Graz Graz Austria; ^6^ Clinical Institute of Medical and Chemical Laboratory Diagnostics University Hospital Graz Graz Austria

## Abstract

Bile salt export pump (*Bsep*) (Abcb11)^*−/−*^ mice are protected from acquired cholestatic injury due to metabolic preconditioning with a hydrophilic bile acid (BA) pool with formation of tetrahydroxylated bile acids (THBAs). We aimed to explore whether loss of *Bsep* and subsequent elevation of THBA levels may have immunomodulatory effects, thus improving liver injury in the multidrug resistance protein 2 (*Mdr2*) *(Abcb4)* ^*−/−*^ mouse. Cholestatic liver injury in *Mdr2* ^*−/−*^ *Bsep* ^*−/−*^ double knockout (DKO), *Mdr2* ^*−/−*^, *Bsep* ^*−/−*^, and wild‐type mice was studied for comparison. *Mdr2* ^*−/−*^ mice were treated with a THBA (3α,6α,7α,12α‐Tetrahydroxycholanoic acid). RNA/protein expression of inflammatory/fibrotic markers were investigated. Serum BA‐profiling was assessed by ultra‐performance liquid chromatography tandem mass spectrometry. Hepatic immune cell profile was quantified by flow cytometric analysis (FACS). *In vitro*, the THBA effect on chenodeoxycholic acid (CDCA)–induced inflammatory signaling in hepatocyte and cholangiocytes as well as lipopolysaccharide (LPS)/interferon‐γ (IFN‐γ)–induced macrophage activation was analyzed. In contrast to *Mdr2* ^*−/−*^, DKO mice showed no features of sclerosing cholangitis. Sixty‐seven percent of serum BAs in DKO mice were polyhydroxylated (mostly THBAs), whereas *Mdr2* ^*−/−*^ mice did not have these BAs. Compared with *Mdr2* ^−/−^, DKO animals were protected from hepatic inflammation/fibrosis. THBA feeding in *Mdr2* ^−/−^ mice improved liver injury. FACS analysis in DKO and *Mdr2* ^−/−^ THBA‐fed mice showed changes of the hepatic immune cell profile towards an anti‐inflammatory pattern. Early growth response 1 (EGR1) protein expression was reduced in DKO and in *Mdr2* ^−/−^ THBA‐fed mice compared with *Mdr2* ^−/−^ control mice. *In vitro*, THBA‐reduced CDCA induced EGR1 protein and mRNA expression of inflammatory markers in hepatocytes and cholangiocytes. LPS/IFN‐γ–induced macrophage activation was ameliorated by THBA. THBAs repress EGR1‐related key pro‐inflammatory pathways. *Conclusion*: THBA and their downstream targets may represent a potential treatment strategy for cholestatic liver diseases.

## Graphical abstract

Loss of *Bsep* (Abcb11) in *Mdr2* (*Abcb4*)^−/−^ mice led to a more hydrophilic, less toxic, bile acid pool mainly consisting of tetrahydroxylated bile acids. Our data demonstrate that tetrahydroxylated bile acids suppress EGR1‐mediated cytokine secretion from hepatocytes, thereby decreasing the recruitment of immune cells such as macrophages and neutrophils to the liver. This immunomodulatory function of tetrahydroxylated bile acids protects *Mdr2* (*Abcb4*)^−/−^ mice from development of hepatic inflammation, fibrosis and bile duct injury.

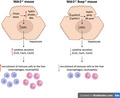

## INTRODUCTION

As a consequence of defective biliary phospholipid secretion and subsequent increase of free nonmicellar bound (potentially toxic) biliary bile acid (BA) concentration, the multidrug resistance protein 2 (*Mdr2*) (*Abcb4*)^−/−^ mouse model of sclerosing cholangitis develops liver and bile duct injury.^[^ ^1^ ^]^ Thereby, this animal model spontaneously develops pericholangitis, ductular proliferation, and onion skin type periductal fibrosis,^[^ ^2^ ^]^ reflecting central morphological features of chronic cholangiopathies, such as primary sclerosing cholangitis (PSC).^[^ ^1^, ^3^ ^]^ Therapeutic options for cholestatic liver disease such as PSC are limited and have so far no established clinical efficacy.^[^ ^3^ ^]^ Ursodeoxycholic acid is the established first‐line treatment for PBC,^[^ ^4^ ^]^ but its efficacy in patients suffering from PSC or cholestatic liver disease based on mutations in the *MDR3* gene (human orthologue of murine *Mdr2*) is limited.^[^ ^5^, ^6^ ^]^ Because changes in bile composition are related to disease progression,^[^ ^7^, ^8^ ^]^ modulation of bile composition appears as an interesting therapeutic strategy.

Absence of bile salt export pump (BSEP/ABCB11) causes severe progressive familial intrahepatic cholestasis type 2 in children that may require liver transplantation or can lead to death.^[^ ^9^, ^10^ ^]^ In contrast to humans, in mice this genetic defect is associated with a milder phenotype lacking the development of progressive cholestasis.^[^ ^11^ ^]^ This observation could—at least in part—be explained by different BA composition, metabolism, and transporter expression in mice and men.^[^ ^11^ ^]^ Importantly, *Bsep* ^*−/−*^ mice develop an adaptive mechanism of BA metabolism and transport, resulting in increased BA hydroxylation/detoxification and increased biliary cholesterol and phospholipid output.^[^ ^12^, ^13^, ^14^ ^]^ Metabolic preconditioning with a hydrophilic BA pool was shown to protect *Bsep* ^*−/−*^ mice from acquired cholestasis.^[^ ^12^ ^]^ Accordingly, absence of BSEP/ABCB11 (resulting in increased hydrophilicity of the intrahepatic BA pool) protects the *Mdr2* ^*−/−*^ mouse model of sclerosing cholangitis from development of liver and bile duct injury.^[^ ^15^ ^]^ However, the underlying mechanisms are still poorly understood. Therefore, the present study was designed to explore whether hydrophilic BAs (especially tetrahydroxylated BAs, as the predominant species in *Bsep* ^*−/−*^ mice) exert immunomodulatory and/or anti‐inflammatory properties, thereby improving liver and bile duct injury in the *Mdr2* ^*−/−*^ mouse model of sclerosing cholangitis.

## MATERIALS AND METHODS

### Animals

*FVB/N Mdr2* ^*−/−*^ mice obtained from Jackson Laboratory (Bar Harbor, ME) were bred with *FVB/N Bsep* ^*−/−*^ mice, obtained from British Columbia Cancer Research Center.^[^ ^11^ ^]^ From the *Mdr2* ^*+/‐*^ *Bsep* ^*+/−*^ colony, all genotypes included in the experiments were bred as littermates. *FVB/N Mdr2* ^*−/−*^ mice were fed with 0.5% 3α,6α,7α,12α‐tetrahydroxycholanoic acid (THBA)–enriched diet for 4 weeks. THBA is commercially available at UHN Shanghai Research & Development, Shanghai, China. All experiments were conducted in 8‐week‐old mice. Animals were housed in a 12‐h light/dark house facility with water and standard chow diet (SSNIFF, Soest, Germany) *ad libitum*. The experimental procedures were approved by the Animal Ethics Committee of the Medical University of Vienna and the Federal Ministry of Science, Research and Economy (BMWFW‐66.009/0008‐WF/3b/2015) and performed according to the Animal Research: Reporting of In Vivo Experiments guidelines.

### Routine serum biochemistry and histology

Serum biochemistry and histological staining (hematoxylin and eosin, sirius red) was performed as described previously.^[^ ^16^ ^]^

### Immunohistochemistry

Detection of hepatic cytokeratin 19 (CK19), F4/80, and MAC‐2 was performed as described previously.^[^ ^17^, ^18^ ^]^ (Antibody information: CK19 antibody [MA5‐15884, Thermo Fisher]; anti‐mouse/human Mac‐2 [Galectin‐3] antibody [CL8942AP, Biocompare]; F4/80 antibody Cl:A3‐1[MCA497R, Bio Rad].)

### Serum BA analysis

Serum BA profiles were acquired using ultra‐performance liquid chromatography tandem mass spectrometry as described previously.^[^ ^19^ ^]^ Levels of polyhydroxylated BAs (> 3 hydroxyl groups) were estimated from peak ion intensities at m/z 423, 439, 530, and 546 for unconjugated and taurine‐conjugated tetrols and pentols, respectively, relative to the internal standard D4‐TCA.

### Messenger RNA analysis and polymerase chain reaction

RNA isolation from liver, complementary DNA synthesis, and real‐time polymerase chain reaction were performed as described previously.^[^ ^20^ ^]^ Oligonucleotide sequences are available upon request.

### Western blot analysis

Protein expression was quantified as described previously.^[^ ^21^ ^]^ Target protein expression was normalized to total loaded protein amount, according to manufacturer's instructions. (Antibody information: EGR1 [44D5] Rabbit mAB #4154, Cell Signaling.)

### Flow cytometric analysis

Livers were dissociated mechanically and digested for 20 min at 37°C using 450 U/ml collagenase I, 125 U/ml collagenase XI, 60 U/ml DNase I, and 60 U/ml hyaluronidase followed by red blood cell lysis (Morphisto, Offenbach am Main, Germany). Samples were passed through 100‐μm strainers before staining. Blocking and staining were performed in phosphate buffered saline supplemented with 2% heat‐inactivated fetal bovine serum (FBS) at 4°C in the dark. For blocking of Fc receptor interactions, 1 × 10^6^ cells were incubated with 2.5 μg/ml of unconjugated anti‐CD16/CD32 antibody (clone 93; eBioscience, Invitrogen). After washing, surface staining was done using the following antibodies: anti‐CD45‐FITC, anti‐Ly6C‐BV605, anti‐Ly6G‐PeCy7, anti‐Cd11b‐AF700, and biotinylated anti‐CD11c (eBioscience, Invitrogen).

### Cell culture

Immortalized human hepatocytes (IHH)^[^ ^22^ ^]^ and murine large bile duct epithelial cells (BECs) were grown in Dulbecco's modified Eagle's medium supplemented with 10% FBS and 1% penicillin/streptomycin. IHHs and BECs were incubated with 200 μM chenodeoxycholic acid (CDCA) and/or 100 μM THBA. After 6 h of treatment, cells were harvested for messenger RNA (mRNA) as well as protein extraction. Experiment was performed two times in triplicates.

The human macrophage cell line THP‐1 cells were seeded at a density of 1.3 × 10^5^ cells per cm^2^ and differentiated using 150 nM phorbol‐12‐myristate‐13‐acetate for 24 h. The THP‐1 cells were rested for a further 24 h in serum containing Roswell Park Memorial Institute 1640 medium. The differentiated THP‐1 cells were stimulated with 10 pg/ml lipopolysaccharide (LPS) and 20 ng/mL interferon‐y (IFN‐γ) in the presence or absence of 100 μM THBA for 24 h.

### Statistical analysis

Results were evaluated using GraphPad Prism version 9.3.1. Statistical analysis was performed using one way analysis of variance. Data were reported as means of 5–7 animals per group ± SD. A *p*‐value < 0.05 was considered significant.

## RESULTS

### Loss of Bsep protects *Mdr2^−/−^* mice from cholestatic liver injury

**FIGURE 1:**
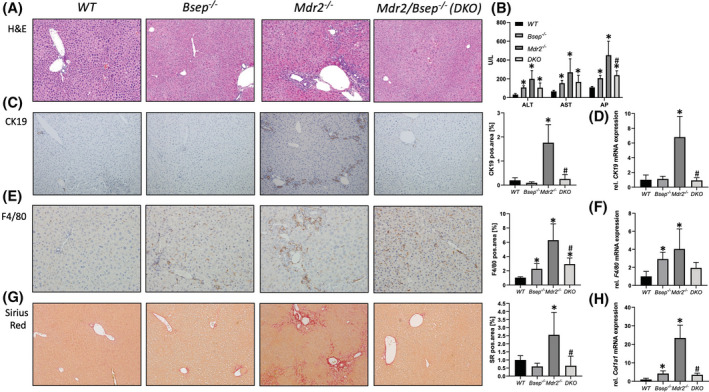
Loss of bile salt export pump (Bsep) improves liver and bile duct injury in the multidrug resistance protein 2 (*Mdr2* ^*−/−*^) mouse model of sclerosing cholangitis. (A) Representative hematoxylin and eosin (H&E) images (×10 magnification) with markedly improved liver histology with reduced pericholangitis and onion skin type of biliary fibrosis in *Mdr2/Bsep* double knockout (DKO) mice. (B) Serum biochemistry reflects unchanged levels of transaminases (alanine aminotransferase [ALT], aspartate aminotransferase [AST]) in *Mdr2/Bsep* DKO compared with single *Mdr2* ^*−/−*^ mice. Alkaline phosphatase (AP) levels are significantly reduced in *Mdr2* ^*−/‐*^ *Bsep* ^*−/−*^ versus *Mdr2* ^*−/−*^ mice. (C) Representative cytokeratin 19 (CK19) immunohistochemistry images (×10 magnification) show reduced cholangiocyte proliferation in *Mdr2/Bsep* DKO mice. (D) Real‐time polymerase chain reaction (PCR) was used to assess the messenger RNA (mRNA) expression of cholangiocyte proliferation marker *CK19*, which was reduced in *Mdr2/Bsep* DKO mice. (E) Representative *F4/80* immunohistochemistry images (×10 magnification) showing reduced numbers of macrophages in the livers of *Mdr2/Bsep* DKO mice. (F) Real‐time PCR was used to assess the mRNA expression of F4/80, as markers of inflammation which was reduced in *Mdr2/Bsep* DKO mice. (G) Representative sirius red (SR) stainings (×10 magnification) show improved biliary fibrosis in *Mdr2/Bsep* DKO. (H) Real‐time PCR was used to assess the mRNA expression of fibrotic marker collagen type I alpha 1 (*Col1a1*), which was reduced in *Mdr2/Bsep* DKO mice. mRNA expression values were normalized against *36b4* levels and are shown relative to expression level in wild‐type (WT) controls. *Significant difference from WT mice; ^#^significant difference from *Mdr2* ^*−/−*^ mice; *p* < 0.05. Computational analysis of histological pictures was done via image J 1.51j8.

Liver histology in *Mdr2*/*Bsep* double knockout (DKO) mice revealed complete reversion of features of sclerosing cholangitis seen in *Mdr2* ^*−/−*^ with pericholangitis and onion skin type of fibrosis (Figure 1A). Accordingly, serum parameters alanine amino transferase (ALT), aspartate aminotransferase (AST), and alkaline phosphatase (ALP) of *Mdr2/Bsep* DKO were significantly reduced compared with *Mdr2* ^*−/−*^ mice (Figure 1B), whereas total serum BA levels were increased in *Bsep* ^*−/−*^ *, Mdr2* ^*−/−*^, and *Mdr2/Bsep* DKO mice compared with wild‐type (WT) control animals (Table 1). Importantly, *Bsep* deficiency reduced bile duct proliferation as determined by immunohistochemistry (IHC) (Figure 1C) and mRNA expression of CK19 (Figure 1D). Because the reactive cholangiocyte phenotype of cholangiopathies is associated with the development of hepatic inflammation and fibrosis, markers for these key processes in liver injury progression were further investigated (Figure 1E–H). F4/80 IHC showed a reduced number of macrophages in livers of *Mdr2/Bsep* DKO mice compared with *Mdr2* ^*−/−*^ mice (Figure 1E). Accordingly, gene‐expression profile of inflammatory marker *F4/80* was markedly reduced in *Mdr2/Bsep* DKO mice (Figure 1F). Sirius red staining (Figure 1G) revealed improvement of fibrosis in *Mdr2/Bsep* DKO mice. Moreover, transcription of fibrotic marker collagen type I alpha 1 (*Col1a1*) was considerably reduced in *Mdr2/Bsep* DKO mice, whereas in *Mdr2* ^*−/−*^ mice the gene expression is increased 23‐fold compared WT control animals (Figure 1H). Together, these data implicate a beneficial effect of loss of *Bsep* on cholestatic liver injury in a mouse model of sclerosing cholangitis (Figure 1).

**TABLE 1:** Total and individual serum bile acid levels

Serum bile acids (μM)	*WT*	*Bsep* ^*−/−*^	*Mdr2* ^*−/−*^	*Mdr2* ^*−/−*^ *Bsep* ^*−/‐*^ *DKO*
TCA	0.26 ± 0.14	3.34 ± 0.60^b^	6.53 ± 1.99^a^	2.11 ± 0.83^a^ ^,^ ^b^
TUDCA	n.d.	0.89 ± 0.86	0.02 ± 0.01	0.33 ± 0.42
TCDCA	0.01 ± 0.00	0.15 ± 0.01^a^	0.06 ± 0.01^a^	0.05 ± 0.02^a^
TDCA	0.03 ± 0.01	n.d.	0.14 ± 0.05^a^	n.d.
CA	0.03 ± 0.05	0.01 ± 0.00	0.03 ± 0.04	0.01 ± 0.00
TοMCA	0.07±	2.26 ± 2.50^a^	0.80 ± 0.25^a^	2.55 ± 0.77^a^ ^,^ ^b^
TαMCA	0.03 ± 0.02	0.58 ± 2.50^a^	0.20 ± 0.05^a^	0.50 ± 0.18^a^ ^,^ ^b^
TβMCA	0.07 ± 0.02	8.67 ± 0.48^a^	2.23 ± 0.34^a^	7.19 ± 3.28^a^ ^,^ ^b^
αMCA	n.d.	0.08 ± 0.48	n.d.	0.04 ± 0.02
οMCA	0.07 ± 0.04	0.36 ± 0.00	0.17 ± 0.05	0.32 ± 0.21
βMCA	0.02 ± 0.04	1.49 ± 0.69	0.04 ± 0.01	0.95 ± 0.89
T‐Tetrols	n.d.	29.21 ± 8.02	n.d.	24.81 ± 12.26
T‐Pentols	n.d.	6.87 ± 1.20	n.d.	7.22 ± 3.93
Tetrols	n.d.	4.55 ± 0.62	n.d.	2.85 ± 1.93
Pentols	n.d.	1.74 ± 0.62	n.d.	1.15 ± 0.62
**Total PHBA**	**n.d.**	**42.37 ± 7.98**	**n.d.**	**36.03 ± 18.45**
**Total BA**	**0.64 ± 0.27**	**60.03 ± 12.03** ^a^	**10.32 ± 2.42** ^a^	**50.14 ± 24.34** ^a^ ^,^ ^b^

Abbreviations: CA, cholic acid; MCA, muricholic acid; PHBA, polyhydroxylated bile acid (>3 hydroxyl groups); TCA, taurocholic acid; TCDCA, taurochenodeoxycholic acid; TDCA, taurodeoxycholic acid; TUDCA, tauroursodeoxycholic acid.

The values in bold are the total PHBA and the total BA levels. While for total BA levels we were able to calculate statistics between all the groups, we could not do that for the total PHBA levels because PHBAs are not present in WT and *Mdr2* KO mice. PHBA levels between *Bsep* KO and *Mdr2*/*BSEP* DKO mice were not statistically different.

^a^ Significant difference from WT mice.

^b^ Significant difference from *Mdr2* ^*‐/‐*^ mice; *p* < 0.05.

### Loss of Bsep alters BA homeostasis and profile in *Mdr2^−/−^* mice

**FIGURE 2:**
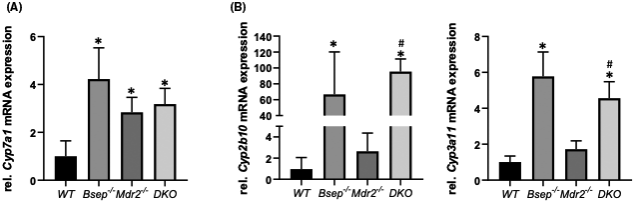
Loss of Bsep results in changes of bile acid homeostasis in the *Mdr2* ^*−/−*^ mouse model of sclerosing cholangitis. (A) mRNA expression levels of *Cyp7a1* are increased in *Bsep* ^*−/−*^, *Mdr2* ^*−/−*^, and *Mdr2/Bsep* DKO mice to the same extent when compared with WT animals. (B) mRNA expression of *Cyp2b10* and *Cyp3a11* are increased in *Bsep* ^*−/−*^ and *Mdr2/Bsep* DKO mice to the same extent. mRNA expression values were normalized against 36b4 levels and are shown relative to expression level in WT controls. *Significant difference from WT mice; ^#^significant difference from *Mdr2* ^*−/−*^ mice; *p* < 0.05.

Next, we investigated whether loss of Bsep in *Mdr2* ^*−/−*^ mice also interferes with BA metabolism and transport. *Cyp7a1* (main enzyme in BA synthesis) was profoundly up‐regulated in *Bsep* ^*−/−*^, *Mdr2* ^*−/−*^, as well as *Mdr2/Bsep* DKO mice (Figure 2A), whereas *Cyp3a11* and *Cyp2b10* (two main enzymes in BA hydroxylation/detoxification) were up‐regulated in *Bsep* ^*−/−*^ as well as in *Mdr2/Bsep* DKO but not in *Mdr2* ^*−/−*^ mice (Figure 2B).

In line with increased BA hydroxylation machinery, about 60%–70% of total serum BAs in *Bsep* ^*−/−*^ and *Mdr2/Bsep* DKO mice were polyhydroxylated (thereby suggested to be less toxic), whereas in serum of *Mdr2* ^*−/−*^ mice the more hydrophobic taurocholic acid (TCA) was the most prominent BA species (Table 1).

### Absence of Bsep with formation of hydrophilic BAs exerts immunomodulatory effects in *Mdr2^−/−^* mice

**FIGURE 3:**
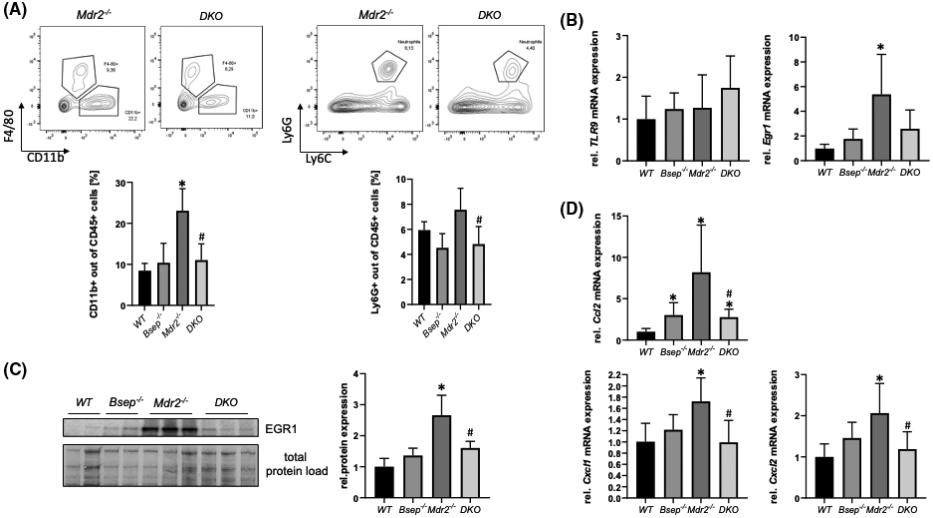
Loss of Bsep exerts immunomodulatory effects in the *Mdr2* ^*−/−*^ mouse model of sclerosing cholangitis. (A) Flow cytometric analysis (FACS) revealed elevated numbers of recruited macrophages (CD11b ^+^ ) and neutrophils (Ly6G ^+^ ) within the CD45^+^ cell population in livers of *Mdr2* ^*−/−*^ mice compared with *WT* mice, whereas in *Mdr2/Bsep* DKO mice these cell populations are in the range of *WT* mice. (B) mRNA expression of toll‐like receptor 9 (*TLR9*) remained unchanged among the different genotypes, while early growth response 1 (*Egr1*) expression levels were increased in *Mdr2* ^*−/−*^ mice compared with *WT* mice. (C) EGR1 protein expression is increased in *Mdr2* ^*−/−*^ mice, whereas it remained unchanged in *Mdr2/Bsep* DKO mice. (D) In line, mRNA expression of EGR1 downstream targets chemokine (C‐C motif) ligand 2 (*Ccl2*), chemokine (C‐X‐C motif) ligand 1 (*Cxcl1*), and *Cxcl2* are reduced *Mdr2/Bsep* DKO mice compared with *Mdr2* ^*−/−*^ mice. Protein data are normalized to total protein and represent means ± SD. mRNA expression data are normalized to *36b4* and are shown relative to untreated control cells. *Significant difference from WT mice; ^#^significant difference from *Mdr2* ^*−/−*^ mice; *p* ≤ 0.05.

To investigate whether changes in BA profile (favoring hydrophilic, less toxic BAs) may exert immunomodulatory, anti‐inflammatory effects, immune cells were isolated from whole liver homogenate. Flow cytometric analysis (FACS) revealed significantly less infiltrating macrophages (CD11b^+^) and neutrophils (Ly6G^+^) in the livers of *Mdr2/Bsep* DKO mice, indicating an improvement of hepatic inflammation compared with *Mdr2* ^*−/−*^mice. Because EGR1^[^ ^23^, ^24^ ^]^ as well as toll‐like receptor 9 (TLR9)^[^ ^25^ ^]^–related pro‐inflammatory pathways are induced via potential toxic BAs such as CDCA and TCA, we explored next whether these inflammatory key players are implicated in the inflammatory liver injury seen in the *Mdr2* ^*−/−*^ mouse model of sclerosing cholangitis. While mRNA of TLR9 remained unchanged among the groups, mRNA as well as protein expression of EGR1 was increased in *Mdr2* ^*−/−*^ mice compared with WT and *Bsep* ^*−/−*^ mice. Notably, EGR1 expression levels in *Mdr2/Bsep* DKO mice were significantly reduced compared with *Mdr2* ^*−/−*^ mice (Figure 3B,C). Accordingly, mRNA expression levels of EGR1 downstream targets such as chemokine (C‐C motif) ligand 2 (*Ccl2*)*, Cxcl1*, and *Cxcl2* were increased in *Mdr2* ^*−/−*^ mice and remained at the level of WT mice in *Mdr2/Bsep* DKO mice (Figure 3D), arguing for anti‐inflammatory properties of THBAs.

### THBA feeding improves liver injury in *Mdr2^−/−^* mice

**FIGURE 4:**
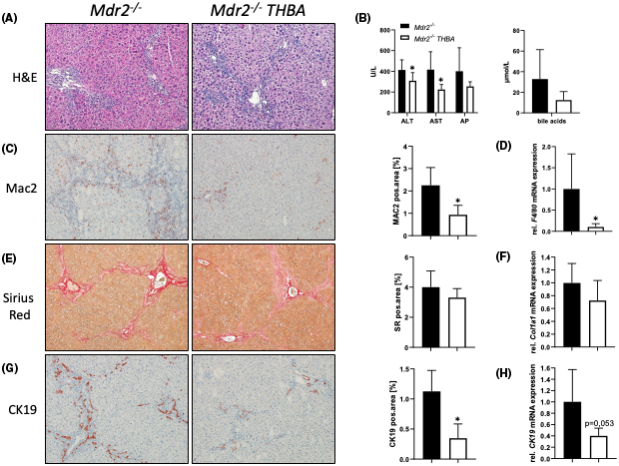
Tetrahydroxylated bile acid (THBA) feeding improves liver and bile duct injury in the *Mdr24* ^*−/−*^ mouse model of sclerosing cholangitis. (A) Representative H&E images (×10 magnification) with improved liver histology in *Mdr2* ^*−/−*^ mice fed a 0.5% wt/wt THBA‐enriched diet for 4 weeks. (B) Serum biochemistry reflects reduced levels of transaminases (ALT, AST) *Mdr2* ^*−/−*^ mice fed 0.5% wt/wt THBA. Total bile acid (BA) levels as well as ALP levels tended to be reduced due to THBA feeding. (C) Representative MAC‐2 immunohistochemistry images (×10 magnification) showing reduced numbers of macrophages in the livers of THBA‐fed *Mdr2* ^*−/−*^ mice. (D) Real‐time PCR was used to assess the mRNA expression of *F4/80*, as markers of inflammation, which was reduced in THBA‐fed *Mdr2* ^*−/−*^ mice. (E) Representative sirius red stainings (×10 magnification) show tendentially reduced biliary fibrosis in *Mdr2* ^*−/−*^ mice fed 0.5% wt/wt THBA. (F) Real‐time PCR was used to assess the mRNA expression of fibrotic marker collagen type I alpha 1 (*Col1a1)*, which was reduced in THBA‐fed *Mdr2* ^*−/−*^ mice. (G) Representative CK19 immunohistochemistry pictures (×10 magnification) show reduced cholangiocyte proliferation in THBA‐fed *Mdr2* ^*−/−*^ mice. (H) Real‐time PCR was used to assess the mRNA expression of cholangiocyte proliferation marker *CK19*, which was reduced in THBA‐fed *Mdr2* ^*−/−*^ mice. mRNA expression values were normalized against *36b4* levels and are shown relative to expression level in WT controls. *Significant difference from *Mdr2* ^*−/−*^ mice; *p* < 0.05. Computational analysis of histological pictures was done via image J 1.51j8.

**FIGURE 5:**
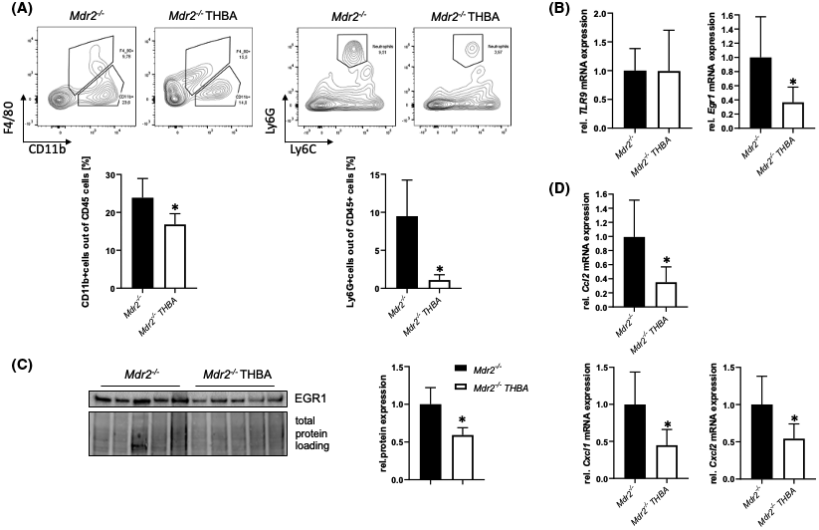
THBA feeding exerts immunomodulatory effects in the *Mdr2* ^*−/−*^ mouse model of sclerosing cholangitis. (A) FACS analysis revealed reduced numbers of recruited macrophages (CD11b ^+^ ) and neutrophils (Ly6G ^+^ ) within the CD45^+^ cell population in livers of *Mdr2* ^*−/−*^ mice fed with 0,5% wt/wt THBA‐enriched diet for 4 weeks compared with untreated *Mdr2* ^*−/−*^ mice. (B) mRNA expression of *TLR9* remained unchanged among the groups, while *Egr1* expression levels were reduced in THBA‐treated *Mdr2* ^*−/−*^ mice. (C) EGR1 protein expression was reduced in *Mdr2* ^*−/−*^ mice fed with THBA. (D) In line, mRNA expression of EGR1 downstream targets *Ccl2, Cxcl1*, and *Cxcl2* was reduced in TBA‐fed *Mdr2* ^*−/−*^ mice. Protein data are normalized to total protein and represent means ± SD. mRNA expression data are normalized to *36b4* and are shown relative to untreated control cells. *Significant difference from WT mice; ^#^significant difference from *Mdr2* ^*−/−*^ mice; *p* ≤ 0.05.

To explore whether the increase of hydrophilic/less toxic BAs in *Mdr2/Bsep* DKO mice was responsible for the improvement of liver and bile duct injury seen in this mouse model, *Mdr2* ^*−/−*^ mice were subjected to 0.5% wt/wt THBA feeding for 4 weeks. While no THBAs were detected in the serum of *Mdr2* ^*−/−*^ control mice, 147 nmol/L THBA was detected in *Mdr2* ^*−/−*^ THBA fed mice, confirming pharmacokinetic efficacy of the used THBA concentration in the diet. Administration of THBA improved liver histology, as well as serum levels of ALT, AST and ALP, and serum BA levels (Figure 4A). Accordingly, liver inflammation, fibrosis, as well as ductular proliferation also improved (Figure 4B–D). Furthermore, FACS analysis demonstrated a significant reduction of infiltrating macrophages (CD11b^+^) as well as neutrophils (Ly6G^+^) in livers or *Mdr2* ^*−/−*^ THBA‐fed mice. Like in the *Mdr2* ^*−/‐*^ *Bsep* ^*−/−*^ mice setting, also under THBA treatment mRNA expression of TLR9 remained unchanged among the groups, while mRNA as well as protein expression of Egr1 was reduced in *Mdr2* ^*−/−*^ THBA fed mice compared with untreated controls (Figure 5B,C). In line, mRNA expression levels of EGR1 downstream targets such as *Ccl2, Cxcl1*, and *Cxcl2* were significantly reduced in *Mdr2* ^*−/−*^ THBA fed mice (Figure 5D).

### THBA treatment ameliorates activation of macrophages *in vitro*

**FIGURE 6:**
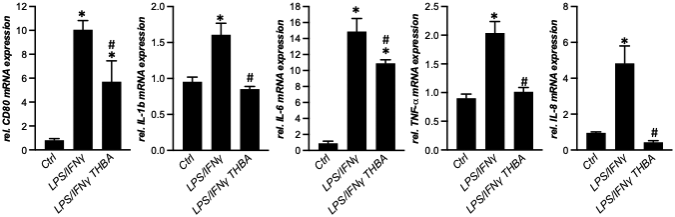
THBA treatment attenuates lipopolysaccharide (LPS)/interferon‐γ (IFN‐γ)–induced macrophage activation *in vitro*. mRNA expression of *CD80*, tumor necrosis factor α (*TNFα)*, interleukin (*IL*)*‐1b, IL‐6*, and *IL‐8* was reduced in THP1 cells with the combination treatment compared with LPS/IFN‐γ monotreatment. mRNA expression data are normalized to *36b4* and are shown relative to untreated control cells. *Significant difference from untreated control cells; ^#^significant difference from LPS/IFN‐γ‐treated cells; *p* ≤ 0.05.

To examine direct immunomodulatory effects of THBA, the human macrophage cell line THP1 was treated with LPS/IFN‐γ for activation (Figure 6). While 24 h of LPS/IFN‐γ treatment increased markers for macrophage activation such as CD80 as well as proinflammatory cytokines such as tumor necrosis factor α (*TNFα*), interleukin (*IL*)*‐1b, IL‐6*, and *IL‐8* combination treatment with 100 μM THBA reduced expression levels (Figure 6).

### THBA treatment attenuates CDCA‐induced EGR1 signaling *in vitro*

**FIGURE 7:**
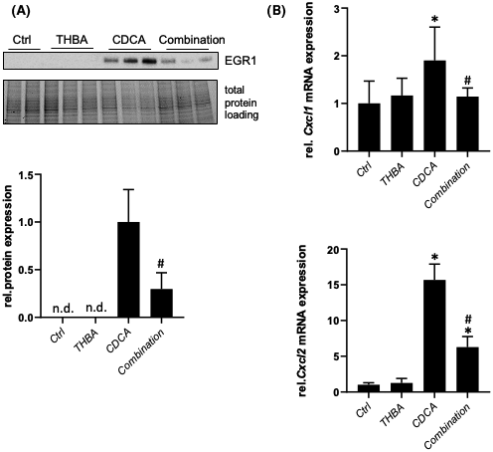
THBA treatment attenuates chenodeoxycholic acid (CDCA)–induced inflammation in hepatocytes *in vitro*. (A) Representative immunoblot and densitometry show that EGR1 protein expression is reduced in IHHs treated with CDCA and THBA for 6 h. (B) In line, mRNA expression of EGR1 downstream targets *Cxcl1* and *Cxcl2* is reduced in the cells with the combination treatment compared with CDCA monotreatment. Protein data are normalized to total protein and represent means ± SD. mRNA expression data are normalized to *36b4* and are shown relative to untreated control cells. *Significant difference from untreated control cells; ^#^significant difference from CDCA treated cells; *p* ≤ 0.05.

To investigate the mechanistic aspects of the observed anti‐inflammatory effect of THBA, IHHs as well as murine large BECs were treated with CDCA as pro‐inflammatory stimulus^[^ ^23^ ^]^ (Figure 7 and Figure S1). Six hours of 200 μM CDCA treatment increased protein expression of EGR1, while combination treatment with 100 μM THBA counteracted EGR1 protein expression in hepatocytes as well as in BECs (Figure 7A and Figure S1A). Accordingly, mRNA expression levels of EGR1 downstream targets such as *Cxcl1* and *Cxcl2* were significantly reduced in the CDCA THBA combination group compared with the CDCA treatment group (Figure 7B and Figure S1B).

## DISCUSSION

Our study demonstrates that *Mdr2* ^*−/−*^ mice lacking Bsep are protected from cholestatic liver and bile duct injury. This protection may at least in part be explained by increased BA hydroxylation resulting in a more hydrophilic (less toxic) BA pool (primarily consisting of THBAs) in the *Mdr2/Bsep* DKO mice (as also seen in the *Bsep* single knock‐out animals^[^ ^12^ ^]^). The reduced numbers of infiltrating monocyte‐derived macrophages and neutrophils in the livers of *Mdr2/Bsep* DKO mice as well as in THBA‐fed *Mdr2* ^*−/−*^ mice, argue for reduced hepatic immune cell recruitment and may implicate a potential immunomodulatory effect of THBAs.

The observation that loss of Bsep and subsequent hydroxylation of the BA pool attenuates cholestatic liver and bile duct injury with inflammation and biliary fibrosis in the *Mdr2* ^*−/−*^ mouse model of sclerosing cholangitis might be attributed to reduced hepatic expression of the pro‐inflammatory key regulator Egr1 (and subsequent downstream targets such as *Cxcl1* and *Cxcl2*) in *Mdr2/Bsep* DKO mice as well as in *Mdr2* ^*−/−*^ mice fed with THBA. This finding is further supported by the *in vitro* perception that THBA treatment improved CDCA‐induced expression of the pro‐inflammatory mediator EGR1 and its downstream targets *Cxcl1* and *Cxcl2*. Because *Cxcl1* and *Cxcl2* are pro‐inflammatory cytokines, which are responsible for immune cell migration,^[^ ^26^ ^]^ their reduced expression/secretion may explain the reduced numbers of infiltrating immune cells seen in THBA‐exposed livers. Thus, the hepatic immune cell profile of DKO as well as THBA‐fed *Mdr2* ^*−/−*^ mice underlines a potential anti‐inflammatory immunomodulatory effect of THBAs.

Notably, exposure of human and mouse hepatocytes to 100 μM TCA increased expression levels of several inflammatory mediators such as cytokines, chemokines, adhesion molecules, and enzymes involved in arachidonic acid metabolism *in vitro*,^[^ ^23^ ^]^ whereas loss of macrophages did not attenuate liver injury in bile duct–ligated mice.^[^ ^27^, ^28^ ^]^ Together with our data, this suggests that not only the reduced immune cell migration but also direct anti‐inflammatory effects of THBA on hepatocytes, cholangiocytes, may contribute to improvement of liver and bile duct injury in DKO as well as in THBA‐fed *Mdr2* ^*−/−*^ mice.

However, despite encouraging anti‐inflammatory effects of THBA, a certain diluting effect of THBA on the hydrophobic intrahepatic BA pool seen in *Mdr2* ^*−/−*^ mice cannot be excluded. Moreover, reduced biliary BA concentration in *Bsep* single knock‐out and *Mdr2/Bsep* DKO could contribute to the more pronounced attenuation of bile duct injury in these animal models.

We could demonstrate an increase in gene‐expression levels of the BA hydroxylating enzymes *Cyp2b10* and *Cyp3a11* in the DKO animals to the same extent like in the *Bsep* ^*−/−*^ mice, whereas in *Mdr2* ^*−/−*^ both enzymes are not affected. The finding that increased levels of THBAs are associated with a protective effect against cholestatic liver disease is in line with what was seen in WT mice subjected to common bile duct ligation after treatment with constitutive androstane receptor and pregnane X receptor agonists regulating the BA hydroxylation enzymes *Cyp2b10* and *Cyp3a11*.^[^ ^29^ ^]^ This observation suggests that DKO animals, like *Bsep* ^*−/−*^ mice, are preconditioned with a hydrophilic BA pool, which protects them from development of cholestatic liver injury.^[^ ^12^ ^]^

Of particular interest, the THBA (3α,6α,7α,12α tetrahydroxycholanoic acid) used in our feeding experiment was detected at high levels in healthy newborns,^[^ ^30^ ^]^ whereas the prognosis of the outcome of infantile intrahepatic cholestasis is very poor in newborns with low levels of THBAs,^[^ ^31^ ^]^ indicating a potential therapeutic function of THBAs in human. Similar to mice, the production of THBA in humans may be part of an overall compensatory mechanism to reduce the level of toxic BAs from hepatocytes. Radiolabeled cholic acid was administered to patients with biliary cirrhosis and severe cholestasis. Five days after administration, about 10% of radioactivity was found as tetrahydroxy‐cholanoates, primarily 3α,6α,7α,12α‐tetrahydroxy‐5β‐ and 1α,3α,7α,12α‐tetrahydroxy‐5β‐cholanoic acids.^[^ ^32^ ^]^ Furthermore, it has been demonstrated that levels of urinary THBA correlated positively with a better clinical outcome in patients with infantile intrahepatic cholestasis,^[^ ^31^ ^]^ arguing for a potential beneficial effect of THBA application to patients with cholestasis.

In conclusion, our study demonstrates that tetrahydroxylated BAs have immunomodulatory effects, shifting the hepatic immune cell profile toward an anti‐inflammatory pattern as well as EGR1 suppressor function. Therefore, our observation may have implications for the human situation by considering tetrahydroxylated BAs as potential therapeutic strategies for pharmacological treatment of patients with cholestasis.

## AUTHOR CONTRIBUTIONS

*Study concept and design*: Claudia D. Fuchs and Michael Trauner. *Manuscript draft*: Claudia D. Fuchs. *Data collection*: Claudia D. Fuchs, Veronika Mlitz, Tim Hendrikx, Annika Wahlström, Marcus Ståhlman, and Michael Trauner. *Statistical analysis*: Claudia D. Fuchs. *Data interpretation*: Veronika Mlitz, Tim Hendrikx, and Annika Wahlström. *Critical revision of the manuscript for important intellectual content*: Marcus Ståhlman, Tatjana Stojakovic, Hubert Scharnagl, Christoph J. Binder, and Hanns‐Ulrich Marschall. *Manuscript outline and revisions, study oversight, and funding acquisition*: Michael Trauner.

## CONFLICT of INTEREST

MT has served as speaker for Falk Foundation, Gilead, Intercept, and MSD; he has advised for Albireo, BiomX, Boehringer Ingelheim, Falk Pharma GmbH, Genfit, Gilead, Intercept, Jannsen, MSD, Novartis, Phenex, Pliant, Regulus, and Shire. He further received travel grants from Abbvie, Falk, Gilead and Intercept, and research grants from Albireo, Alnylam, Cymabay, Falk, Gilead, Intercept, MSD Takeda, and UltraGenyx. He is also co‐inventor of patents on the medical use of NorUDCA filed by the Medical Universities of Graz and Vienna. HUM served as consultant for Albireo, AstraZeneca, Amylyx, Bayer, Calliditas, Intercept, Inorbit, Mirum and Zealand, and received travel grants from Falk. CF received travel grants from Gilead, Roche, Falk, Merck, Vifor, Abbvie, and Böhringer Ingelheim. All other authors have no financial disclosures concerning this study to report.

## Supporting information


**Figure S1** Tetrahydroxylated bile acid (THBA) treatment attenuates chenodeoxycholic acid (CDCA)–induced inflammation in cholangiocytes *in vitro*. (A) Representative immunoblot and densitometry. Early growth response 1 (EGR1) protein expression is reduced in murine large bile duct epithelial cells (BECs) treated with CDCA and THBA for 6 h. (B) Messenger RNA (mRNA) expression of EGR1 downstream targets of chemokine (C‐X‐C motif) ligand 1 (*Cxcl1*) and *Cxcl2* are reduced in the cells with the combination treatment compared with CDCA monotreatment. Protein data are normalized to total protein and represent means ± SD. The mRNA expression data are normalized to *36b4* and are shown relative to untreated control cells. *Significant difference from untreated control cells; ^#^Significant difference from CDCA‐treated cells (*p* ≤ 0.05)
