# Supplementary figures and images for: Tetrahydroxylated bile acids improve cholestatic liver and bile duct injury in the Mdr2−/− mouse model of sclerosing cholangitis via immunomodulatory effects
Source: Hepatol Commun. 2022 Jun 12;6(9):2368–78. doi: 10.1002/hep4.1998 (PMC9426398; doi:10.1002/hep4.1998)

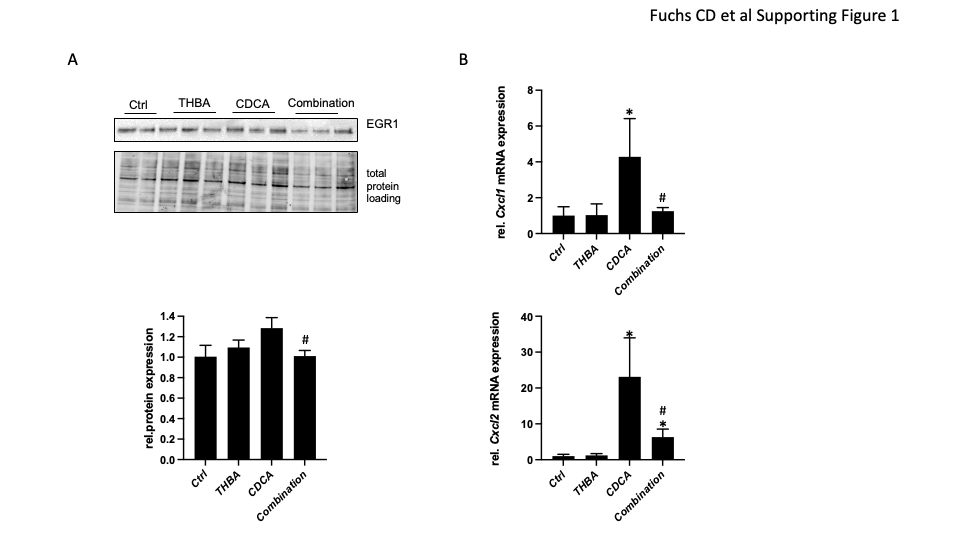

Supplement: Supplementary file 1 — Figure S1 Tetrahydroxylated bile acid (THBA) treatment attenuates chenodeoxycholic acid (CDCA)–induced inflammation in cholangiocytes in vitro. (A) Representative immunoblot and densitometry. Early growth response 1 (EGR1) protein expression is reduced in murine large bile duct epithelial cells (BECs) treated with CDCA and THBA for 6 h. (B) Messenger RNA (mRNA) expression of EGR1 downstream targets of chemokine (C‐X‐C motif) ligand 1 (Cxcl1) and Cxcl2 are reduced in the cells with the combination treatment compared with CDCA monotreatment. Protein data are normalized to total protein and represent means ± SD. The mRNA expression data are normalized to 36b4 and are shown relative to untreated control cells. *Significant difference from untreated control cells; #Significant difference from CDCA‐treated cells (p ≤ 0.05) [file HEP4-6-2368-s001.tif]
